# Long-Term Total Neoadjuvant Therapy Leads to Impressive Response Rates in Rectal Cancer: Results of a German Single-Center Cohort

**DOI:** 10.3390/curroncol30060407

**Published:** 2023-05-31

**Authors:** Georg W. Wurschi, Stefan Knippen, Thomas Ernst, Claus Schneider, Herry Helfritzsch, Henning Mothes, Yves Liebe, Martin Huber, Andrea Wittig

**Affiliations:** 1 Clinician Scientist Program, Interdisciplinary Center for Clinical Research (IZKF), Department of Radiotherapy and Radiation Oncology, Jena University Hospital, 07747 Jena, Germany; 2 Department of Radiotherapy and Radiation Oncology, Jena University Hospital, 07747 Jena, Germany; 3 University Tumor Center (UTC), Jena University Hospital, 07747 Jena, Germany; 4 Department of General, Visceral and Vascular Surgery, Jena University Hospital, 07747 Jena, Germany; 5 Department of General, Visceral and Thoracic Surgery, Thuringia-Clinic Saalfeld Georgius Agricola, 07318 Saalfeld, Germany; 6 Department of General, Visceral and Vascular Surgery, Sophien-und Hufeland-Klinikum Weimar, 99425 Weimar, Germany; 7 Department of General and Visceral Surgery, SRH Wald-Klinikum Gera, 07548 Gera, Germany; 8 Department of General, Visceral and Vascular Surgery, Robert-Koch-Hospital, 99510 Apolda, Germany

**Keywords:** locally advanced rectal cancer, FOLFOX4, TNT, consolidation, preoperative chemoradiation, toxicity

## Abstract

Intensified preoperative chemotherapy after (chemo)radiotherapy, (Total Neoadjuvant Therapy–TNT), increases pathological complete response (pCR) rates and local control. In cases of clinically complete response (cCR) and close follow-up, non-operative management (NOM) is feasible. We report early outcomes and toxicities of a long-term TNT regime in a single-center cohort. Fifteen consecutive patients with distal or middle-third locally advanced rectal cancer (UICC stage II–III) were investigated, who received neoadjuvant chemoradiotherapy (total adsorbed dose: 50.4 Gy in 28 fractions and two concomitant courses 5-fluorouracil (250 mg/m^2^/d)/oxaliplatin (50 mg/m^2^), followed by consolidating chemotherapy (nine courses of FOLFOX4). NOM was offered if staging revealed cCR 2 months after TNT, with resection performed otherwise. The primary endpoint was complete response (pCR + cCR). Treatment-related side effects were quantified for up two years after TNT. Ten patients achieved cCR, of whom five opted for NOM. Ten patients (five cCR and five non-cCR) underwent surgery, with pCR confirmed in the five patients with cCR. The main toxicities comprised leukocytopenia (13/15), fatigue (12/15) and polyneuropathy (11/15). The most relevant CTC °III + IV events were leukocytopenia (4/15), neutropenia (2/15) and diarrhea (1/15). The long-term TNT regime resulted in promising response rates that are higher than the response rates of short TNT regimes. Overall tolerability and toxicity were comparable with the results of prospective trials.

## 1. Introduction

Until recently, the standard of care for locally advanced rectal cancer (LARC, UICC stage II and III) comprised neoadjuvant chemoradiotherapy (CRT) followed by resection and adjuvant chemotherapy (CTx). Recent trials have demonstrated the beneficial effects of intensified preoperative CTx regarding pathological complete response (pCR) rates and progression-free survival (PFS) [1,2,3,4,5]. The so-called “total neoadjuvant therapy” (TNT, intensified preoperative treatment) often comprises concomitant 5-Fluorouracil (5-FU)- and oxaliplatin-based chemoradiotherapy (CRT) regimes in combination with 3–6 cycles of fluoropyrimidine-based consolidating CTx [1,2]. Alternatively, less intensive concomitant CRT protocols (including 5-FU monotherapy) were followed by nine, more intense cycles of FOLFOX [3]. A non-operative approach (NOM) appears to be safe in cases of clinical complete response (cCR) in highly selected cases [5,6], translating into a gain in quality of life (QoL) compared to mandatory surgery and frequent colostomy [7,8].

The cumulative CTx dose in current TNT protocols is lower than the cumulative dose patients received in the intervention group of the ACO/ARO/AIO-04 trial [9]. This protocol was applied regularly at our institution before TNT was introduced. A pre-operative CTx with similar doses (Preoperative, concomitant CRT: oxaliplatin 50 mg/sqm d1, 8, 22, 29; 5-FU 250 mg/sqm/d d1–14 + d22–35) was followed by adjuvant CTx for eight cycles. Oxaliplatin was dosed higher (100 mg/sqm, q2w for eight cycles) than currently common for consolidation CTx of current TNT protocols (mostly 50–85 mg/sqm, q2w for up to nine cycles). However, long-term data beyond three years’ follow-up are not yet available for current TNT protocols. So, this de-escalation compared to adjuvant CTx protocols should be further investigated regarding long-term overall survival (OS) and progression-free survival (PFS).

In a subanalysis of the results of the ARO-04 trial [9], Hofheinz et al. demonstrated that, in particular, patients below 60 years of age benefited from adding oxaliplatin through lower local and systemic recurrence rates and even improved overall survival [10]. However, there are only a few prospective trials, such as the ADORE trial [11], demonstrating the benefits on disease-free survival (DFS) through adding oxaliplatin to adjuvant CTx. A Cochrane Review of further trials comparing the outcome of pre- and postoperative CTx [12] and the EORTC 22921 trial [13] indicated that the combination of pre- and postoperative CTx might not improve survival. Lim et al. demonstrated that, especially, patients obtaining pCR after preoperative CRT do not significantly benefit from adjuvant CTx [14].

**Figure 1:**
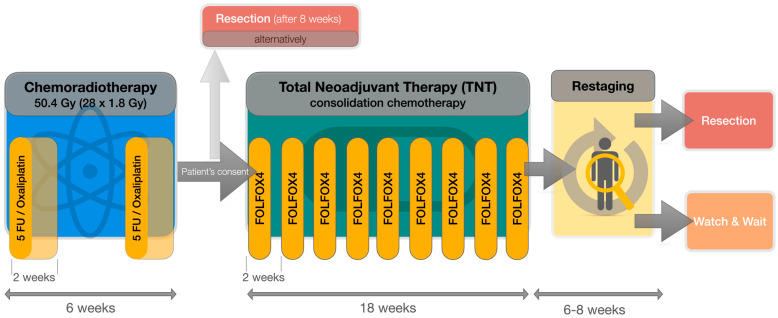
Flowchart of the applied total neoadjuvant therapy (TNT) protocol, consisting of simultaneous chemoradiotherapy (CRT) with 5-Fluorouracil (5-FU, 250 mg/sqm/d continuously d1–14 and d22–35) and Oxaliplatin (50 mg/sqm on d1, d8 + d21, d28), followed by consolidation chemotherapy with FOLFOX4 (Oxaliplatin 85 mg/sqm on d1; 5-FU 400 mg/sqm d1 + d2; continuous 5-FU 1200 mg/sqm/d d1–2, folinic acid 200 mg/sqm d1 + d2) every 2 weeks (q2w).

Thus, intensification of neoadjuvant treatment by CTx appears to be beneficial compared to adjuvant CTx. Nevertheless, TNT seems to be a demanding therapy and not suitable for every patient due to treatment-related side effects. We, therefore, suppose a tolerability-guided two-step protocol to maximize patient benefits while simultaneously avoiding undertreatment in patients not tolerating consolidating CTx. This protocol combines long-term CRT, which is followed by consolidating CTx in cases of good tolerability of CRT and has been routinely applied at our institution for three years (Figure 1). This work is intended to report on first experiences with long-term consolidating CTx by this protocol in a single-center cohort and to compare these early results with other TNT protocols.

## 2. Materials and Methods

We analyzed 15 consecutive patients who received TNT between March 2020 and April 2022. In accordance with the current recommendation of the German colorectal tumor expert associations (ACO, AIO, ARO) [15,16], patients with LARC received TNT if at least one of the following criteria was fulfilled: T3-tumors with distal location and/or positive lymph nodes (N+), additional risk factors (lateral positive pelvic lymph nodes, extramural vascular invasion-EMVI, infiltration of mesorectal plane-CRM+), or any T4-tumor (Table 1).

Our institutional standard comprises simultaneous CRT with two cycles of 5-FU (250 mg/sqm/d continuously d1–14 and d22–35) and Oxaliplatin (50 mg/sqm on d1, d8 + d21, d28) analogous to the ACO/ARO/AIO-04 protocol [9] (Figure 1). All patients with LARC were undergoing reevaluation at the end of CRT regarding general health condition, treatment-related side effects and patient consent for consolidation CTx (“TNT” regime). Good general health condition (Karnofsky Performance Score, KPS, ≥70%), as well as good compliance, was mandatory for TNT. Patients not fulfilling these criteria underwent routine surgery 6 to 8 weeks after CRT. Adjuvant CTx was recommended to these patients in cases with risk factors, such as unfavorable pTNM assessment following excision.

To allow maximization of tumor regression and further benefits of neoadjuvant therapy, we prefer a longer preoperative interval comprising up to nine cycles of consolidation CTx in case of good tolerance. FOLFOX4 (oxaliplatin 85 mg/sqm on d1; 5-FU 400 mg/sqm d1 + d2; continuous 5-FU 1200 mg/sqm/d d1–2, folinic acid 200 mg/sqm d1 + d2) was chosen for consolidation CTx. The cumulative CTx doses of this protocol range between the less-intense ARO-12 protocol [1] and the, far higher-dosed, PRODIGE-23 protocol [4]. It is comparable with the RAPIDO- [3] and OPRA protocols [5], providing a slightly higher cumulative oxaliplatin dose but less 5-FU overall. Furthermore, the overall cumulated CTx dose of this protocol is similar to the doses patients received by neoadjuvant and adjuvant treatment in the intervention arm of the ACO/AIO/ARO-04 trial.

The CTx was delivered according to the prescribing information of the single agencies and dose reductions were performed if clinically indicated. Hematological toxicity, liver or kidney impairment as well as general condition and neuropathy were considered as measures for dose reduction.

All patients were required to undergo a preoperative restaging 4 to 6 weeks after TNT, including rectoscopy and magnetic resonance imaging for local response assessment, as well as computed tomography for distant metastasis detection. Upon the recommendation of the multidisciplinary tumor board, NOM was offered to all patients obtaining cCR willing to undergo close follow-up. Standard resection (e.g., total mesorectal excision, TME) was conducted in all other cases 6 to 8 weeks after TNT and pCR rates were reported.

This two-step neoadjuvant CRT/CTx-design allows individual therapy adaptions during routine CRT without undertreatment if no additional consolidation CTx (“TNT”) was performed, as this therapy is still equivalent to the established ARO-04 protocol. It simultaneously allows therapy intensification by performing TNT in selected cases (see Table 1 for indications).

General health condition, blood counts and treatment-related side effects (according to EORTC CTCAE v5.0) were monitored weekly during CRT as well as prior to each cycle of consolidating CTx and were analyzed retrospectively for this study. We will further report on early outcomes, such as response rates after TNT and DFS as well.

The early results of this protocol are reported in this article. Due to the small sample size (*N* = 15) and limited observation period, we focus on descriptive analyzes. IBM SPSS Statistics version 29 (IBM Deutschland GmbH, Ehningen, Germany) was used for statistics.

## 3. Results

We identified 53 patients with rectal cancer who underwent neoadjuvant CRT between March 2020 and April 2022. Within this group, 15 patients with LARC received TNT and met our eligibility criteria for this analysis (see also Appendix A Figure A2). We included nine male and six female patients with ages ranging between 53 and 79 years (mean: 66.4 years ± 5.84 SD). These patients were followed-up for at least 9 months (mean 18 months, min. 9 months, max. 24 months). The mean follow-up for patients undergoing NOM was 17.4 months (min. 9 months, max. 24 months).

The tumors were mostly staged T3 N+ with additional risk factors (12/15 patients) and located distally (8/15 patients). Table 2 shows the detailed demographic characteristics of the cohort.

The TNT regime was near-completely applied in 11/15 patients (i.e., application of at least 8 cycles of FOLFOX4), corresponding to a 73.3% completion rate (Table 3).

A dose reduction of CTx was required often due to hematological toxicity or polyneuropathy. Only 3/15 patients received all cycles of FOLFOX4 without dose reduction. On average, five cycles of FOLFOX4 were administered in the scheduled dose. The total cumulative oxaliplatin dose of concomitant and consolidation CTx was 844.6 mg/m^2^ (±66.8 SD) on average. Termination of consolidation CTx was necessary in 3/15 patients. The reasons were impaired general health condition (2 patients) or hematological toxicity (1 patient).

**Figure 2:**
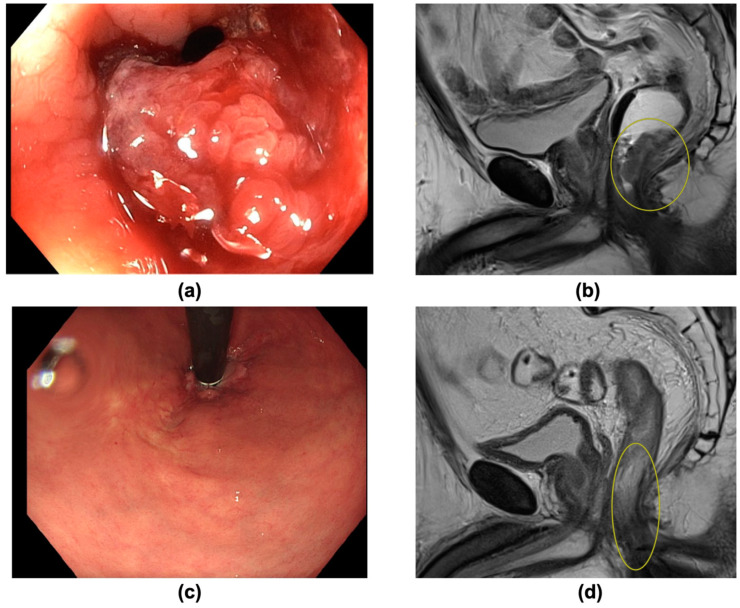
(**a**–**d**): A 63-year-old patient with distal rectal carcinoma (cT4 cN0 cM0) was undergoing Total Neoadjuvant Therapy (TNT), obtaining clinical complete response (cCR). A non-operative management was chosen. There was no recurrence within an 18-month follow-up. (**a**) Image of endoscopy prior to treatment, showing a vulnerable ulcerated tumor. (**b**) Pre-treatment sagittal T2-weighted MRI scan, picturing a distal T4 tumor (yellow circle) in minimal distance from the anal sphincter. (**c**) Endoscopy 8 weeks after TNT, showing a macroscopic good response with a residual scar. (**d**) Post-treatment sagittal T2-weighted MRI scan confirming clinical complete response 8 weeks after TNT (yellow circle: former tumor bed).

The majority of patients underwent resection (10/15) with a pCR rate of 50% (5/10). Five patients (5/15) with cCR underwent NOM (“watch and wait”). An exemplary patient, opting for NOM after obtaining cCR, is shown in Figure 2. In total, a cumulative complete response (cCR or/and pCR) was obtained in 10/15 patients (66.7%).

A relapse was diagnosed in two patients. One patient developed multiple distant metastases after 12 months after resection and was lost to follow-up after 24 months. Another patient, undergoing NOM, was diagnosed with liver metastases 24 months after TNT. No local recurrence was reported.

**Figure A1:**
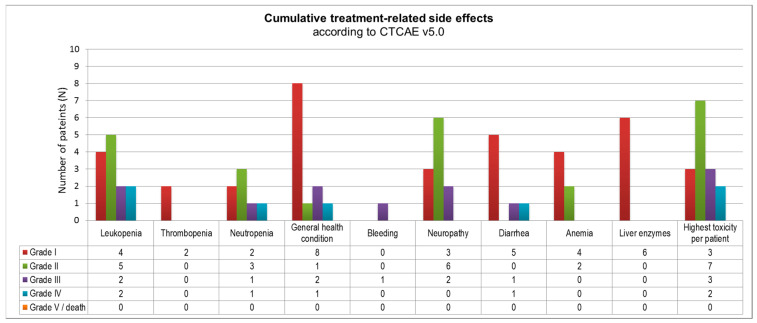
Overview of reported cumulative toxicities according to EORTC CTCAE v5.0. Overall, Grade 3 or 4 toxicity was reported in 5/15 patients (last column).

**Figure A2:**
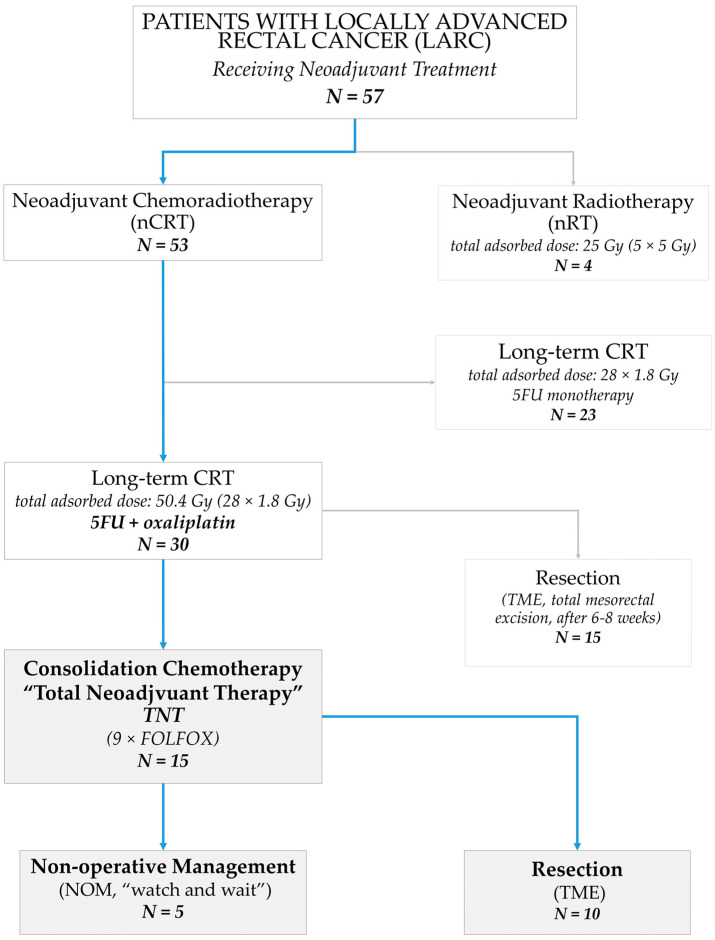
Study profile showing all patients who received a neoadjuvant treatment for locally advanced rectal cancer (LARC) at our institution between March 2020 and April 2022. The subgroup comprising all patients who underwent consolidation chemotherapy, i.e., total neoadjuvant therapy (TNT), is described in this work.

The most common treatment-related side effects of sequential CTx were hematological toxicity and polyneuropathy, as well as impaired general health condition (i.e., fatigue), as summarized in Table 4 and Figure A1. Among these, hematological toxicity was reported most often. Leukocytopenia was observed in 13/15 patients (86.7%), thereof nine patients did not exceed common toxicity criteria grade (CTC °I–II). Anemia and elevated liver enzymes occurred in six patients respectively, never affecting subsequent therapy (CTC °I–II). Thrombocytopenia occurred seldom (2/15 patients).

Overall, CTC grade I or II events were reported in 10/15 patients and CTC grade III or IV events occurred in 5/15 patients. No CTC grade V events were reported.

Therapy limitations were mostly due to hematological toxicity. At least one course was not administered in 4/15 patients. A high rate of any grade of polyneuropathy was observed (11 patients/73.3%), but only two patients developed severe symptoms (CTC °III). One therapy discontinuation was due to simultaneous febrile neutropenia and severe diarrhea (CTC °IV) after two courses; however, the patient recovered completely and underwent resection. Other discontinuations were related to a severe SARS-CoV-2 infection in a single patient, so resection after reconvalescence was favored, and lacking compliance in another patient.

We report postoperative complications in 3/10 patients undergoing resection. An anastomotic insufficiency, delayed wound healing with secondary infection and a vaginal fistula occurred in one patient each. TNT was terminated after two cycles of FOLFOX in the patient who developed the vaginal fistula due to hematological toxicity. In the other two patients, TNT was administered as scheduled.

**Figure 3:**
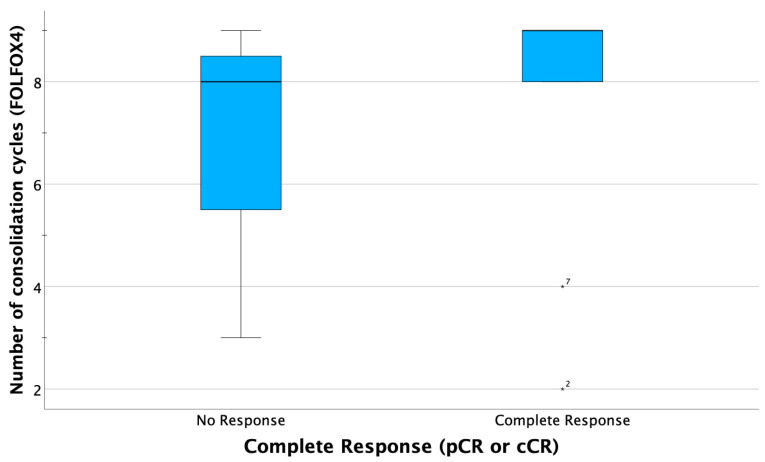
Boxplots comparing summarized complete response (CR) rates (clinical CR in case of organ preservation and pathological CR in case of resection), indicating that most patients obtaining CR received at least eight courses consolidating chemotherapy. In two patients (ID 2 and ID7), complete response was observed after only 2 or 4 cycles FOLFOX, respectively.

**Figure 4:**
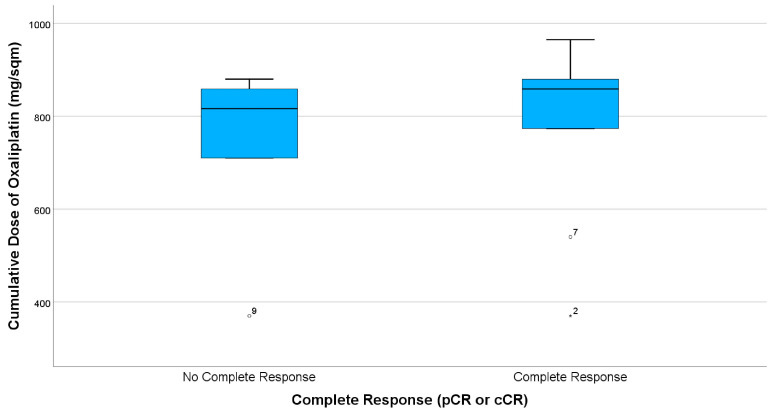
Boxplots comparing summarized complete response (CR) rates (i.e., clinical CR in case of organ preservation and pathological CR in case of resection), indicating that most patients obtaining CR received cumulative Oxaliplatin doses of more than 774 mg/sqm. There are three patients, having only received two cycles (ID2, ID9) or four cycles (ID7) FOLFOX, respectively.

As displayed in the paired boxplots (Figure 3 and Figure 4), complete response was mostly observed in patients receiving at least eight cycles of consolidating chemotherapy or accordingly cumulative oxaliplatin doses of more than 774 mg/sqm. However, testing for a correlation of higher oxaliplatin doses with complete response is not possible due to the small sample size.

## 4. Discussion

Despite the limitations of the small sample size of this retrospective analysis, we are able to report encouraging response rates. Earlier trials evaluating TNT protocols commonly reported the benefit of consolidation CTx compared to induction CTx [1,5]. We, therefore, focused on comparing consolidation CTx regimes of the following studies.

The RAPIDO trial [3] performed nine courses of FOLFOX4 as well, but in total, it was less intense due to the use of short-course radiotherapy only. The regimen resulted in similar therapy-related side effects to our protocol. However, in particular, CTC °I–II neuropathy was slightly higher (79%) in the RAPIDO trial. Overall, pCR rates were substantially lower (28%). However, there is limited comparability between both groups as more patients with high risk factors or more invasive (i.e., T4) tumors were included in the RAPIDO trial.

Another quite intense protocol was applied in the experimental arm of PRODIGE-23 [4]: an induction CTx with six courses of FOLFIRINOX continued by six courses of mFOLFOX6 postoperatively was applied. Despite the limited comparability due to different treatment sequences, PRODIGE-23 resulted in higher cumulative CTx doses and seemed to have caused more toxicities: CTC °I–II neuropathy was higher (83%) and severe diarrhea was reported more often (CTC °I–II: 62%, CTC °III–IV: 11%). Hematological toxicity was within the same range (CTC °I–II about 40–50%, summarized).

The ARO-12 trial [1] was based on the same treatment sequence, comprising simultaneous CRT followed by a shorter consolidation CTX with three cycles of FOLFOX in the experimental arm. Complete response was reported in 25%, but the shorter interval between CRT and resection limits comparisons. Nevertheless, neurological and hematological toxicity were lower, possibly due to less intense consolidation CTx.

The more recent data of the OPRA trial suggested higher response rates compared to our cohort: the approach was similar with long-course CRT (infusional 5-FU monotherapy) followed by eight cycles of FOLFOX4. The majority of the patients received NOM (experimental arm: 120/158 patients, 76%), meaning clinical complete response was achieved after TNT. The cumulative rate of CTC °III–IV events was reported as 38%, but unfortunately, not further specified. This is comparable with the treatment-related side effects observed in our cohort (CTC °III + IV in 5/15 patients–33.3%), suggesting reproducible tolerability of these quite similar protocols.

Another recent trial, ARO-16 [2], investigated intended organ preservation. A similar treatment to the ARO-12 trial was performed, resulting in 15% cCR after 15 weeks according to first results. The cCR rate increased up to 40% after 28 weeks, supporting the hypothesis that a longer preoperative (consolidation) period might be beneficial regarding response rates. CTC °III–IV toxicity was slightly higher than in our cohort (45%).

CR rates were within the same range of longer-lasting consolidation protocols, such as the OPRA trial [5]. We reported higher toxicity compared to less-intense, shorter protocols (such as ARO-12 [1]), but saw higher response rates. It seems that longer-lasting neoadjuvant protocols, comprising simultaneous conventional fractionated CRT and TNT for at least 4 months, have the potential to maximize tumor regression. The tolerability of these protocols and our TNT regime were comparable, but increased long-term therapy-related side effects, such as polyneuropathy, have to be considered and closely monitored. However, to maintain an adequate tolerability in our cohort, dose reductions had to be performed often and only a small percentage of patients received the scheduled dose. The small sample size does not allow us to conclude whether the high CR rates were attributable to a high cumulative chemotherapy dose or the long neoadjuvant period. This aspect needs to be further investigated in order to evaluate the possibility and safety of de-escalating the chemotherapy in order to reduce toxicity, which is probably mostly related to high cumulative oxaliplatin doses. We saw a high rate of polyneuropathy, as patients in this cohort often received cumulative oxaliplatin doses of more than 750 mg/sqm, which is associated with a higher prevalence of polyneuropathy [17,18].

If adjuvant CTx would be indicated instead of consolidating CTx, comparable or even higher cumulative CTx doses would be administered. Consequently, higher toxicity was reported by trials reporting on adjuvant therapy as the standard of care [11,12,19]. These findings emphasize the importance of risk stratification upfront in TNT to avoid overtherapy as well as close monitoring of therapy-related side effects for consecutive individual dose adaptions [20].

The promising early results reported here need to be validated through additional data to obtain a statistically significant cohort. We are currently performing a local pilot study, applying this TNT regime (PRIMO Trial, NCT 05524012) [21]. Multimodal diagnostic, comprising, e.g., multiparametric magnetic resonance imaging (MRI) and liquid biopsy, will be used to quantify tumor response longitudinally in the PRIMO trial. Furthermore, long-term results are necessary to assess the effectiveness of this treatment intensification regarding PFS and OS.

## 5. Conclusions

The long-term TNT protocol, consisting of conventional fractionated long-course CRT and 4 months of consolidation CTx, seems to maximize tumor regression and response rates.

We experienced comparable tolerability of this regime and promising response rates compared to other TNT protocols. Nevertheless, the application of nine cycles of FOLFOX4 is a demanding therapy, leading to relevant hematological toxicity or neuropathy in a subset of patients. This requires a frequent evaluation of side effects with treatment adaptation. Long-term outcomes and a greater cohort size are needed to validate these early data.

## Figures and Tables

**Table 1:** Patient characteristics and tumor stages.

Patient Characteristics	15 Patients					
Mean age	66.4 years (±5.84 SD)					
	Minimum	53 years	Maximum	79 years		
Gender (*N*/%)	Female	6/40%	Male (*N*/%)	9/60%		
**Tumor Characteristics**						
Tumor localization	Distal (0–6 cm)	8/53.3%	Medium (6–12 cm)	7/46.7%	High (>12 cm)	0/0%
Clinical T category	T3	12/80%	T4	3/20%		
Clinical N category	N0	2/13.3%	N1	6/40%	N2	7/46.7%

T3 N0 and T3 N1 tumors were only included in case of additional risk factors or distal location. Risk factors: positive lateral pelvic nodes; EMVI—extramural vascular invasion; CRM+—infiltration of mesorectal plane; SD—standard deviation.

**Table 2:** Indications for Total Neoadjuvant Therapy (TNT) in this case series.

Criteria for Total Neoadjuvant Therapy (TNT) According to Institutional Standard	
LARC, UICC stage II/III	Each T4 Tumor; Each T3 tumor when located distally (0–6 cm) T3 and location > 6 cm: in case of risk factors (e.g., N+, EMVI+, CRM+, >cT3b)
Sufficient liver/renal function; No severe cytopenia	GFR > 45 mL/min; liver enzymes < 2.5 ULN Neutrophil counts ≥ 3 × 10^9^/L; Thrombocyte counts ≥ 100 × 10^9^/L; Hemoglobin ≥ 6 mmol/L
Adequate general health condition	Karnofsky Performance Score (KPS) ≥ 70%
No further relevant secondary diagnosis	No homozygous DPD-deficiency No severe cardiac disease ^#^ No severe pulmonary disease ^§^ No severe psychologic disorder

Abbreviations: CRM±infiltration of the mesorectal plane, EMVI—extramural vascular invasion, BMI—body mass index, GFR—glomerular filtration rate, Karnofsky performance score—KPS, N+ positive lymph nodes, ULN—upper limit of normal range. ^#^ Severe cardiac disease includes clinical relevant heart failure according to NYHA III–IV, cardiac arrhythmia that requires intervention and/or history of acute coronary syndrome (ACS) within the 6 months prior to treatment. ^§^ Severe pulmonary disease includes severe obstructive lung diseases (COPD GOLD III–IV) and/or other pulmonary diseases requiring hospitalization within the 6 months prior to treatment.

**Table 3:** Main outcome features.

*N* Total	15 Patients		
**Treatment procedure**			
	Resection (TME)	10	66.7%
	pCR after TME	5	33.3%
	NOM (“watch and wait”)	5	33.3%
**Completion of consolidating chemotherapy**			
Yes *(8/9x FOLFOX4)*		11	73.3%
No/Discontinuation	Total	4	26.7%
*Reasons for discontinuation*	KPS alteration *	2	13.3%
	Hemotoxicity *	1	6.7%
	SARS-CoV-2 infection	1	6.7%
	Compliance	1	6.7%
	Diarrhea *	1	6.7%

*N*_(total)_ = 15 patients; Completion of TNT is defined as successful application of at least 8 courses of FOLFOX4. NOM—non-operative management, TME—total mesorectal excision, cCR—clinical complete response, pCR—pathological complete response; TNT—total neoadjuvant therapy; KPS—Karnofsky Performance score; * One dropout was due to simultaneous hematological toxicity and KPS alteration after severe diarrhea.

**Table 4:** Overview of most relevant therapy-related side effects, according to EORTC-CTCAE v5.0. “Liver enzymes” comprises ALAT, ASAT, gamma-GT, Bilirubin and AP levels, the highest of which defined the CTC grade. * Including febrile neutropenia.

Toxicity (EORTC-CTCAE v5.0)	CTC °I		CTC °II		CTC °III		CTC °IV		CTC °V	Total	
	*N*	%	*N*	%	*N*	%	*N*	%	*N*	*N*	%
Polyneuropathy	3	20.0	6	40.0	2	13.3	-	0	0	11	73.3
Diarrhea	5	33.3	1	6.7	1	6.7	1	6.7	0	7	46.7
Liver enzymes	6	40.0	-	0	-	0	-	0	0	6	40.0
Hemoglobin	4	26.7	2	13.3	-	0.0	-	0	0	6	40.0
Leukocytopenia	4	26.7	5	33.3	2	13.3	2	13.3	0	13	86.7
Neutropenia	2	13.3	3	20.0	1	6.7	1 *	6.7	0	7	46.7
Thrombocytopenia	2	13.3	-	0	-	0	-	0	0	2	13.3
Fatigue/General health condition	8	53.3	1	6.7	2	13.3	1	6.67	0	12	80.0

## Data Availability

The datasets generated and analyzed during the current study will be not publicly available due to local data privacy restrictions but anonymized data will be available from the corresponding author after completion of recruitment on reasonable request.

## Abbreviations

ACO	Arbeitsgemeinschaft Chirurgische Onkologie of DKG (Surgical Oncology working group of German Cancer Society)
AIO	Arbeitsgemeinschaft Internistische Onkologie of DKG (Medical Oncology working group of German Cancer Society)
ALAT	alanine transaminase
AP	alkaline phosphatase
ARO	Arbeitsgemeinschaft Radiologische Onkologie of DKG (Radiation Oncology working group of German Cancer Society)
ASAT	aspartate transaminase
BMI	body mass index
cCR	clinical complete response
COPD	chronic obstructive pulmonary disease
CRM+	infiltration of mesorectal plane
CRT	chemoradiotherapy
CT	computed tomography
CTC	common toxicity criteria
CTx	chemotherapy
d	days (since beginning of Radiotherapy/Chemotherapy)
DFS	Disease-free survival
EMVI+	extramural vascular invasion
gGT	gamma-glutamyl transferase
GFR	glomerular filtration rate
GOLD	Global Initiative for Chronic Obstructive Lung Disease
KPS	Karnofsky’s Performance Status
LARC	locally advanced rectal cancer
MRI	magnetic resonance imaging
N	positive lymph nodes
NOM	Non-operative management (“watch and wait”)
NYHA	New York Heart Association
OS	overall survival
pCR	pathological complete response
QoL	quality of life
q2w	once every 2 weeks
PFS	Progression-free survival
RT	radiotherapy
SD	standard deviation
sqm	square meter (reference on body surface area)
TME	total mesorectal excision
TNT	total neoadjuvant therapy
UICC	Union Internationale Contre Le Cancer
ULN	upper limit of normal range
